# Very high coronary artery calcium score with normal myocardial perfusion SPECT imaging is associated with a moderate incidence of severe coronary artery disease

**DOI:** 10.1007/s00259-015-3072-z

**Published:** 2015-07-04

**Authors:** Salem A. Yuoness, Ahmed M. Goha, Jonathan G. Romsa, Cigdem Akincioglu, James C. Warrington, Sudip Datta, David R. Massel, Rafael Martell, Sanjay Gambhir, Jean-Luc C. Urbain, William C. Vezina

**Affiliations:** Department of Nuclear Medicine, London Health Sciences Centre, 800 Commissioners Road East, PO Box 5010, London, ON N6A 5W9 Canada; Division of Cardiology, London Health Sciences Centre, London, Ontario Canada; Private Practice, London, Ontario Canada

**Keywords:** CT coronary artery calcium score, Myocardial perfusion imaging, Sestamibi

## Abstract

**Purpose:**

Myocardial perfusion imaging (MPI) has limitations in the presence of balanced multivessel disease (MVD) and left main (LM) coronary artery disease, occasionally resulting in false-normal results despite the high cardiovascular risk associated with this condition. The purpose of this study was to assess the incidence of severe coronary artery disease (CAD) in the presence of a very high Agatston coronary artery calcium (CAC) score (>1,000) in stable symptomatic patients without known CAD but with normal MPI results.

**Methods:**

A total of 2,659 prospectively acquired consecutive patients were referred for MPI and evaluation of CAC score by CT. Of this patient population, 8 % (222/2,659) had ischemia without myocardial infarction (MI) on MPIand 11 % (298/2,659) had abnormal MPI (MI and/or ischemia). On presentation 1 % of the patients (26/2,659) were symptomatic, had a CAC score >1,000 and normal MPI results. The definition of normal MPI was strict and included a normal hemodynamic response without ischemic ECG changes and normal imaging, particularly absence of transient ischemic dilation. All of these 26 patients with a CAC score >1,000 and normal MPI findings underwent cardiac catheterization.

**Results:**

Of these 26 patients, 58 % (15/26) had severe disease (≥70 % stenosis) leading to revascularization. Of this group, 47 % (7/15) underwent percutaneous intervention, and 53 % (8/15) underwent coronary artery bypass grafting. All of these 15 patients had either MVD (14/15) or LM coronary artery disease (1/15), and represented 0.6 % (15/2,659) of all referred patients (95 % CI 0.3 – 0.9 %). The majority, 90 % (8/9), had severe CAD with typical chest pain.

**Conclusion:**

A very high CAC score (>1,000) with normal MPI in a small subset of symptomatically stable patients was associated with a moderate incidence of severe CAD (95 % CI 37 – 77 %). Larger studies and/or a meta-analysis of small studies are needed to more precisely estimate the incidence of CAD in this population. This study also supports the concept that a normal MPI result in patients with severe CAD may be due to balanced MVD.

**Electronic supplementary material:**

The online version of this article (doi:10.1007/s00259-015-3072-z) contains supplementary material, which is available to authorized users.

## Introduction

Patients with normal myocardial perfusion imaging (MPI) results on SPECT have a very low cardiac event rate estimated at <1 % per year [1–3]. Although rare, left main (LM) coronary artery disease, and balanced multivessel disease (MVD) can result in a falsely normal MPI study despite the high associated cardiovascular risk [4, 5]. Only a fraction of patients with coronary artery disease (CAD) involving the LM artery or MVD have perfusion abnormalities in all the coronary artery territories on MPI [6]. Clinical information, exercise data and other stress parameters provide important additional information to identify these patients [4, 7–12].

Atherosclerosis may be associated with calcium deposition in the coronary arteries [13]. Coronary calcification can be quantified on CT using the Agatston coronary artery calcium (CAC) score (see the CAC bibliography, that includes reviews, in Appendix 1 of the Electronic supplementary material). CAC scoring has been used in asymptomatic individuals to diagnose atherosclerosis and to predict future cardiac events, i.e., to determine the long-term 10-year mortality rate beyond the Framingham risk score. High CAC scores are associated with a greater degree of stenosis and ischemia. The distribution of CAC scores in patients with normal MPI is wide.

Our hypothesis was that a very high CAC score of >1,000 with normal MPI, in the absence of high-risk markers, particularly transient ischemic dilation (TID), would be associated with severe MVD.

## Materials and methods

### Patient population

**Fig. 1:**
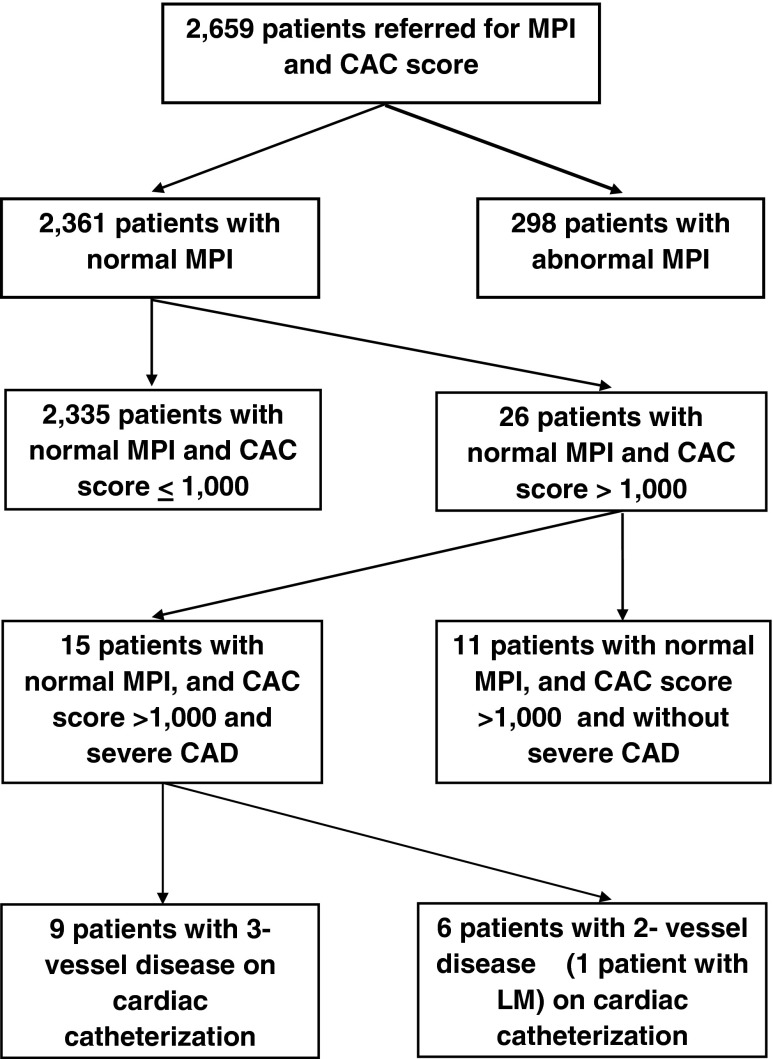
Flow chart of patient enrollment. *MPI* myocardial perfusion imaging, *CAC score* Agatston coronary artery calcium score, *CAD* coronary artery disease, *LM* left main coronary artery disease

We prospectively evaluated 2,659 consecutive patients, referred by internists and cardiologists for clinical reasons, for a hybrid study involving both MPI and CAC scoring between January 2007 and July 2011 (Fig. 1).

Our study was approved by our local university ethics board. All patients signed written informed consent to be entered into a registry and be followed clinically. The consent did not include an invasive coronary angiography (ICA). The patients completed a written cardiac questionnaire and were interviewed by a cardiology nurse and a nuclear cardiology physician. Historical evaluation included: previous cardiac testing, procedures, and consultation reports which were reviewed by a Nuclear Medicine fellow and a Cardiology fellow. The characteristics of the 2,659 patients referred are presented in detail in Appendix 2 of the Electronic supplementary material.

We found 26 patients without known CAD with normal MPI and a CAC score of >1,000 and without high-risk parameters, particularly absence of transient TID. High risk parameters did not include the CAC score, an independent variable. The demographics of the 26 patients with normal MPI and a CAC score of >1,000 are shown in Table 1. The pretest probability of CAD was determined using the model of Diamond et al. [14].

**Table 1:** Demographics of the 26 patients with coronary artery calcium score > 1,000

	All patients (*n* = 26)	Patients with severe CAD (*n* = 15)	Patients without severe CAD (*n* = 11)
Male	17 (65 %)	10 (38 %)	7 (27 %)
Age (years)			
Mean ± SD	68 ± 10	70 ± 10	65 ± 10
Range	40 – 81	53 – 81	40 – 74
Hypertension	18 (69 %)	9 (35 %)	9 (35 %)
Diabetes	5 (19 %)	2 (8 %)	3 (12 %)
Dyslipidemia	13 (50 %)	8 (31 %)	5 (19 %)
Family history	6 (23 %)	5 (19 %)	1 (4 %)
Smoking	7 (27 %)	3 (12 %)	4 (15 %)
Pretest probability of CAD			
Low	2 (8 %)	1 (4 %)	1 (4 %)
Intermediate	15 (58 %)	6 (23 %)	9 (35 %)
High	9 (35 %)	8 (31 %)	1 (4 %)
Reasons for referral			
Chest pain	19 (73 %)		
Typical	9 (35 %)	8 (31 %)	1 (4 %)
Atypical	10 (38 %)	4 (15 %)	6 (23 %)
Chest pain and/or (subjective or objective) dyspnea	4 (15 %)	3 (12 %)	1 (4 %)
Preoperative assessment with chest pain	2 (8 %)	0	2 (8 %)
LBBB with atypical chest pain	1 (4 %)	0	1 (4 %)

Values are number (%) of patients, except age in years

*CAD* coronary artery disease, *LBBB* left bundle branch block

Physician-supervised maximum-workload symptom-limited exercise [15] was performed if possible, with or without pharmacological vasodilation. Target heart rate was not a criterion for stopping exercise.

### Myocardial imaging

For details of the technique see Appendix 3 of the Electronic supplementary material (Myocardial Imaging Technique) which follow the American Society of Nuclear Cardiology guidelines [16]. The definition of normal MPI was strict (Appendix 4 of the Electronic supplementary material: Definition of a Normal MPI [17–21]). In summary, normal MPI included normal images with normal hemodynamic response and without ischemia on ECG, and absence of TID (the normal TID ratio is <1.20).

### Coronary calcium score

The Agatston CAC scores were obtained on a GE LightSpeed VCT 64-detector CT scanner within 1 week of the stress MPI study and analyzed using an AW 4.4 workstation [22]. A very high CAC score was defined as >1,000. This value has been used in multiple studies to define a very high CAC score in asymptomatic individuals to predict future cardiac events beyond the Framingham risk score [2, 22, 23]. CAC scores were acquired and analyzed by one experienced technologist using prospective ECG gating, a 2.5-mm slice thickness, 120 kV tube voltage, 200 mAs tube current per rotation, and a large field-of-view of 50 × 50 cm. The CAC score was calculated with commercially available software (Smartscore, GE Healthcare) [21].

### Coronary angiography

We prospectively encouraged these symptomatic patients with normal MPI and a very high CAC score of >1,000 to undergo ICA, as our hypothesis was that a clinically significant incidence of severe CAD would be present in this population. All 26 patients underwent ICA. A visual grading system was used to assess the severity of lesions in all major coronary arteries. Stenosis was defined as: no stenosis (0 – 24 %), mild (25 – 49 %), moderate (50 – 69 %) and severe 70 – 100 %), except for LM coronary artery disease where moderate stenosis was 25 – 49 % and severe stenosis was 50 % or greater [24]. Catheterization was performed using Siemens Axiom Artis equipment. The scoring system reflects the most severe stenosis in each major vessel, i.e. the LM coronary artery (we assigned to two-vessel disease), the left anterior descending coronary artery, the left circumflex coronary artery, and the right coronary artery, or their major branch vessels >1.5 mm in diameter). The intermediate ramus branch was defined as a branch of the left anterior descending coronary artery by convention.

### Statistical methods

Statistical analysis was performed with commercially available software (SPSS version 16.0), and confirmed with previously available RS/1 software (BBN technologies). Categorical variables are presented as frequencies and continuous variables as means ± SD for normal distributions and medians ± interquartile ranges (IQRs) for skewed distributions. Variables were compared with Fisher’s exact test for categorical variables and by Student‘s unpaired *t* test for continuous variables, unless otherwise specified. Two-tailed *P* values <0.05 were considered statistically significant.

## Results

### Demographics of patients with CAC score >1,000 and normal MPI

The demographics of the 26 symptomatic patients without known CAD with normal MPI results and a CAC score of >1,000 are shown in Table 1.

### CAC scores

The mean CAC score in the 26 patients was 2,131 (range 1,104 – 5,194). The mean CAC score in the 15 patients with severe CAD was 2,229 (range 1,106 – 5,194), and the mean CAC score in the 11 patients without severe CAD was 2,093 (range 1,104 – 3,585; *P* = 0.58, not significant). Thus the CAC scores were similar.

### Left ventricular ejection fraction

The median left ventricular ejection fraction (LVEF) in the 15 patients with severe CAD (≥70 % stenosis) was 65 % (IQR 5 %), and the median LVEF in the 11 patients without severe CAD was also 65 % (IQR 5 %; *P* = 0.99, not significant).

### Coronary angiography

Table 2 shows the ICA results in all 26 patients including 9 patients with normal coronary arteries or mild CAD and 17 patients with moderate to severe CAD. Of the 17 patients with moderate to severe CAD, approximately two-thirds (ten patients, nine with severe CAD) had three-vessel disease, approximately one-third (six patients, all with severe CAD) had two-vessel disease (LM coronary artery disease was categorized as two-vessel disease), and one patient with moderate CAD had single-vessel disease. Thus 15 patients (58 %) had severe CAD (≥70 % stenosis). None of the patients with severe CAD (*n* = 15) had single-vessel disease, 6 (40 %) had two-vessel disease and 9 (60 %) had three-vessel disease.

**Table 2:** Coronary angiography results in all 26 patients

	Moderate–severe CAD (*N* = 17, 65 %)	Normal–mild CAD (*N* = 9, 35 %)
Normal	–	1 (11 %)
Mild (<50 % stenosis)	–	8 (90 %)
Moderate (50 – 69 % stenosis)	2 (12 %)	–
Severe (≥70 % stenosis)	15 (88 %)	–
Normal vessels	–	1 (11 %)
Single-vessel disease	1 (6 %)	1 (11 %)
Two-vessel disease	6 (35 %)	3 (33 %)
Three-vessel disease	10 (59 %)	4 (44 %)

*CAD* coronary artery disease

### Percutaneous coronary intervention/coronary artery bypass grafting

Of the 15 patients with severe CAD, 7 (47 %) underwent stenting and 8 (54 %) underwent coronary artery bypass grafting.

### Number of CAD risk factors

The patients with and without severe stenosis had, on average, two CAD risk factors. The number of CAD risk factors in 15 patients with severe CAD was 1.8 ± 1.3 and in 11 patients without severe CAD was 2.0 ± 1.2 (*P* = 0.69, not significant).

### Typical chest pain

Of the 26 patients, 17 had atypical chest pain and 9 had typical chest pain. As shown in Table 3, typical chest pain occurred in 8 of the 15 patients with severe CAD and was significantly more frequent in those with than in those without severe CAD (*P* = 0.04) being present in only 1 (9 %) of the 11 patients without severe CAD. Thus the incidence of severe CAD was 90 % (8 of 9 patients) among those with typical chest pain with a normal MPI result and CAC score >1,000.

**Table 3:** Typical chest pain

	Yes	No
Patients with severe CAD (*n* = 15)	8 (53 %)*	7 (47 %)
Patients without severe CAD (*n* = 11)	1 (9 %)	10 (91 %)

*P = 0.04 with severe CAD and typical chest pain

Severe CAD: ≥70 % stenosis

### Stress testing

Pharmacological vasodilator stress alone was performed in the minority of patients (Table 4). Only 1 of the 15 patients with a falsely normal MPI result and severe obstructive CAD (≥70 % stenosis) underwent pharmacological vasodilator stress without supplementary exercise.

**Table 4:** Stress testing

	Combined (exercise + pharmacological)	Treadmill exercise	Pharmacological
With severe CAD (*n* = 15)	13 (87 %; cycling 11, treadmill 2)	1 (7 %)	1 (7 %)
Without severe CAD (*n* = 11)	7 (64 %; cycling 6, treadmill 1)	1 (9 %)	3 (27 %)

*P* = 0.40, not significant (Freeman-Halton extension of the Fisher’s Exact test)

Exercise was maximum workload symptom-limited, plus pharmacological vasodilator stress if needed

### Index case

**Fig. 2:**
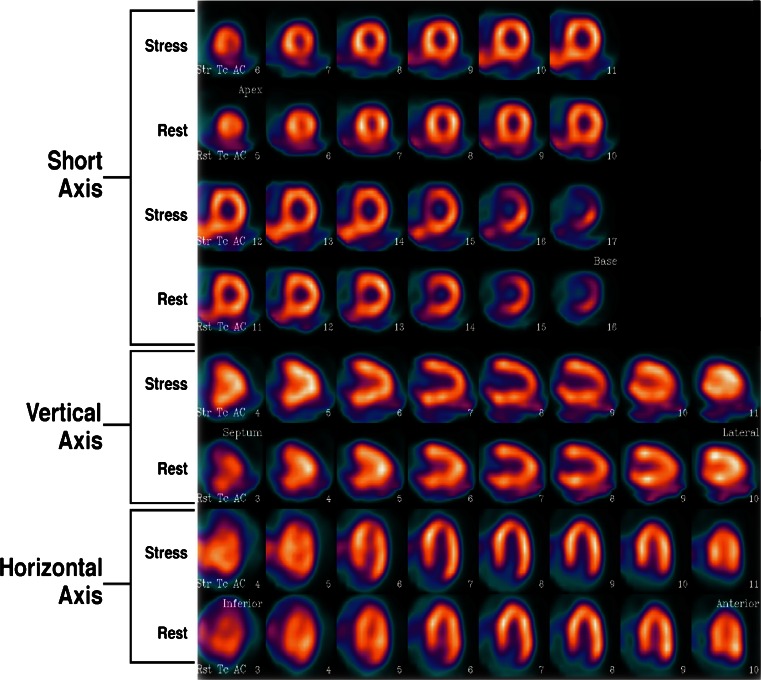
Two-day (rest/stress) MPI study using half dose in a patient with typical chest pain. Attenuation-corrected (*AC*) images with iterative reconstruction and depth-dependent resolution recovery (stress images above and corresponding rest images below) do not show a perfusion defect. The images without attenuation correction (not shown) had a normal transient dilation ratio of 1.15 (normal <1.20). Attenuation artifacts disappeared with attenuation correction. These attenuation corrected images with depth resolution recovery show possible transient dilation ratio visually. Normal range for transient dilation ratio with attenuation correction is not known

**Fig. 3:**
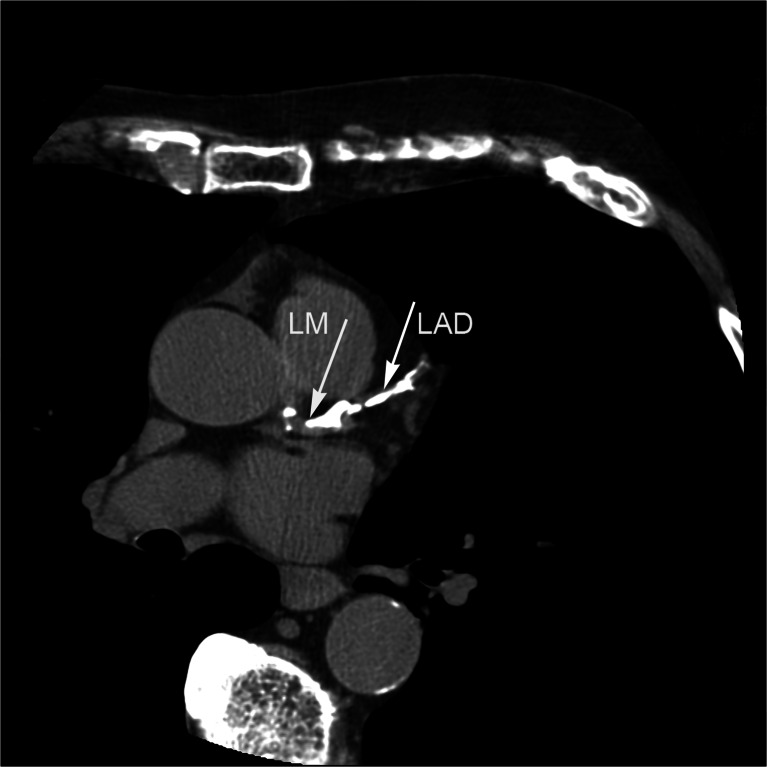
Dense coronary artery calcification in the left main coronary artery (*LM*) distally and in the left anterior descending coronary artery (*LAD*). The coronary artery calcium score was 4,024. *Arrows* indicate the mid-LM coronary artery and the proximal LAD coronary artery

**Fig. 4:**
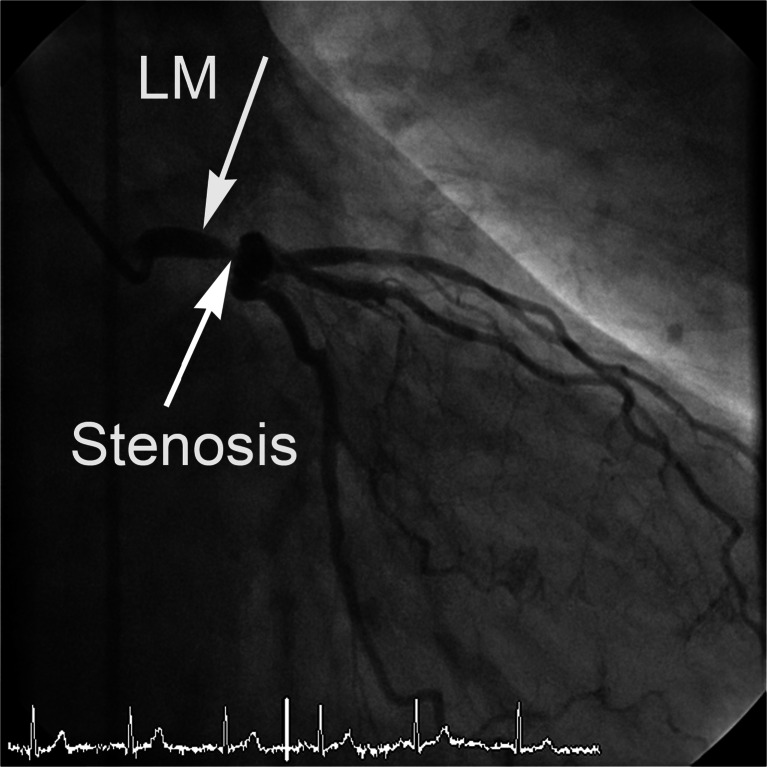
Selective coronary angiogram with catheter in the left main coronary artery (*LM*). Severe stenosis with tapering of the left main coronary artery distally and with poststenotic dilation (aneurysm) are seen. *Arrows* indicate the mid-LM coronary artery and tight stenosis of the LM coronary artery distally

A 79-year-old man (178 cm, 85 kg) had typical chest pain, hypertension and dyslipidemia. Two-day MPI was performed using half-time acquisition. Dipyridamole plus gentle cycling (50 W) was used for stress, with 1,000 MBq of sestamibi for rest and stress imaging and iterative reconstruction with attenuation correction and depth resolution recovery (Fig. 2). This showed homogeneous perfusion with questionable TID visually. The left ventricle did not appear larger after stress, either visually (not shown) or quantitatively using filtered back projection reconstruction (TID 1.15; normal <1.20). The CAC score was 4,024 (Fig. 3). ICA showed a severe stenosis in the left main coronary artery distally with poststenotic dilation (aneurysm formation; Fig. 4).

## Discussion

Our study showed a moderate incidence of severe obstructive multivessel CAD in all 26 symptomatic patients without known CAD with a normal MPI result and a CAC score of >1,000. All underwent ICA. The other patients with normal MPI who had a CAC score <1,000 are shown in Appendix 5 of the Electronic supplementary material. This group appeared to have a lower pretest probability of CAD as expected as shown in in Appendix 6 of the Electronic supplementary material. Ischemia is more closely related to the absolute CAC score than CAC percentile scores adjusted for age, ethnicity, and gender [25], as is prognosis. We used an absolute CAC score.

False-negative MPI studies in patients with LM coronary artery disease and/or MVD is a limitation of MPI. In one study, without accounting for high-risk nonperfusion variables, LM coronary artery disease and/or MVD were missed in 17 % of patients with normal MPI [4]. The disease was not missed in any patient when high-risk variables were considered, particularly TID. Many of these patients (45 %, 45/101) underwent exercise stress testing alone. In another study, 58 consecutive patients with a normal MPI result underwent ICA [5]. Of these 58 patients, 18 had 30 stenoses (>50 % stenosis). Logistic regression analysis revealed typical angina to have the strongest association. Typical angina in patients referred for noninvasive imaging has recently been shown [26, 27] to have a lower predictive value than originally proposed by Diamond et al. [14]. The work of Diamond et al. was based on patients interviewed by a cardiologist, and was not based on self-administered cardiac questionnaires, and all the patients underwent ICA. In our study, 90 % of the patients (eight of nine) with typical chest pain, a CAC score >1,000, and normal MPI, had severe CAD (stenosis ≥70 %). By combining high-risk nonperfusion variables, particularly TID, clinical variables, particularly typical angina, and a very high CAC score, the likelihood of false-negative MPI studies may be reduced.

Our study expands the limited literature (two papers) angiographically demonstrating CAD in patients with normal MPI, and a high CAC score [28, 29]. Choudhary et al. [28] examined a small number of patients (*n* = 84) with normal MPI and a high CAC score. CAD was documented on coronary CT angiography (CCTA), but not on ICA. The rate of abnormalities among our patients was low, although greater than among the patients in the study by Choudhary et al. In our study, 11 % of patients (298/2,659) had an abnormal MPI result. In the study by Choudhary et al., 4 % of patients (3/84) had an MPI abnormality (less robust). Choudhary et al. chose a lower CAC score of >400. The other study of false-negative MPI results in patients with a very high CAC score of >1,000 was by Ghadri et al. [29] whose patients underwent ICA. Single-vessel disease was more prevalent in the study by Ghadri et al. being seen in 41 % [15/29] of patients with a false-negative MPI result and a CAC score >1,000, whereas none of our patients, who had more severe stenosis (≥70 %) and normal MPI, had single-vessel disease. The studies by Ghadri et al. and Choudhary et al. differ from our study in that stenosis was defined visually as 50 % or greater, whereas we chose 70 % or greater, except in LM coronary artery disease where we chose 50 % or greater. The false-negative rate found by Choudhary et al. and Ghadri et al. might have been related to the lack of exercise with vasodilator pharmacological stress. Choudhary et al. used dipyridamole in some of their patients, without exercise. Ghadri et al. used , Rozanski et al. [31] assessed the frequency of cardiac death and myocardial infarction over a mean follow-up of 32 ± 16 months in 1,153 patients undergoing both CAC scanning and MPI. Of these patients, 140 (28 ischemic, 112 nonischemic) had a CAC score >1,000. The authors excluded revascularized patients (10 of the 112 nonischemic patients underwent early revascularization at <60 days or late revascularization at >60 days), in contrast to our study in which such patients were included. They suggested that high CAC scores do not confer an increased risk of cardiac events. The excluded group of patients who underwent revascularization (9 % of non-ischemic patients) might have been at greater risk of cardiac death/myocardial infarction events. The true number of patients with severe CAD could be greater as not all patients with a CAC score >1,000 underwent ICA. This is partial verification bias. Follow-up with at least 80 % success only occurred for 2 years (50 % follow-up at 3 years). Therefore, prognosis assessment was short-term.

### Study strengths

All patients in our study completed a self-administered cardiac questionnaire and were interviewed by a cardiology nurse and a nuclear cardiologist. Data were recorded using a predesigned form specifically designed to support a research database. The large-scale CAC score studies obtained patient information solely from patient-completed cardiac questionnaires [2, 26]. By performing MPI and assessing CAC simultaneously, we reduced a source of referral bias to CAC scoring [32]. Maximum-workload symptom-limited exercise was performed whenever possible in addition to pharmacological stress. All patients had ICA. This eliminated partial verification bias (posttest referral bias). The “gold standard” in our study was ICA, not CCTA. Multiple studies by highly experienced readers at respected academic institutions have shown lower diagnostic accuracy of 64-slice CCTA compared with ICA, particularly in patients with CAC scores above 400 [33–36]. CCTA often requires aggressive pretreatment with beta blockers to achieve diagnostic quality images whereas ICA does not.

### Study limitations

Our study had a limited selection of physician-referred patients, as do all single-center studies. Dyspnea was entered into the database, whether subjective or objective, whether expected (e.g. pulmonary disease) or due to other disease, or due to advanced age, deconditioning or unexpected disease (possible angina equivalence). Thus, the incidence of dyspnea was high in our referred population. We investigated a very small subgroup. This subgroup would have been larger if our selection criteria had been broader (we strictly defined “normal” MPI to avoid the possibility of including patients with any abnormality or possible abnormality). However, we wanted to determine the incremental value of one variable. We used visual assessment of the degree of stenosis on ICA. We did not use functional flow reserve (FFR) [37]. A coronary stenosis apparent visually may not be hemodynamically significant.

Comparison of the severity of coronary artery stenosis on ICA and functional severity as assessed using FFR showed that 35 % of 50 – 70 % stenoses and 80 % of 71 – 90 % stenoses were functionally significant and almost all substantially occluded arteries (96 %) were significantly affected [37]; clearly stenosis of 70 % or greater correlates better with functional assessment than stenosis of 50 % or greater. The lack of FFR assessment in our study would probably have had no effect as 15 of 26 patients (58 %) had severe disease, and only 2 patients (7 %) with moderate disease might have benefited from FFR.

We used SPECT MPI rather than quantitative PET; the latter is better at detecting balanced disease [9]. Quantitative three-dimensional myocardial blood flow imaging with PET is not as available as SPECT. The incidence of false-negative MPI results could have been greater in our 2,659 patients than we have reported, since we only examined patients with a CAC score of >1,000. Dyspnea was noted in 37 % (973/2,659) of our referred patients. Dyspnea includes subjective (patient declared) and physician determined objective dyspnea not explained by pulmonary disease (angina equivalence).

### Clinical applicability, generalizability and clinical relevance

CT attenuation-corrected MPI requires an emission and a transmission scan. A single transmission scan can be used for CT attenuation correction of the rest and stress MPI emission scans. The CAC score can be used for CT attenuation correction of SPECT and PET images [38]. Thus, the CAC score can be obtained without another transmission scan acquisition. There are vendors currently offering traditional sodium iodide crystal/photomultiplier tube Anger systems with an integrated multislice diagnostic CT scanner, e.g. a six-slice CT scanner. The newer solid-state cadmium-zinc-telluride (CZT) gamma cameras may not have integrated attenuation correction capability. Currently a 64-slice CT system integrated with a CZT camera is commercially available. CZT MPI studies without an integrated diagnostic CT scanner require a separate acquisition for a transmission map either using a low-end CT scanner, not capable of obtaining a CAC score, or using a separate stand-alone CT scanner capable of obtaining a CAC score.

The importance of perfusion versus anatomy (CCTA/ICA) in risk stratification is under investigation. MPI currently remains an important widely available method for risk stratification, particularly in older patients. TID is lauded as a clinically useful marker of severe CAD [20]. This was based on treadmill exercise MPI without attenuation correction with greater than 85 % heart rate. However, two recent studies in which stress was not limited to adequate treadmill exercise demonstrated low yield and nonspecifically of TID [39, 40]. Another recent study has also shown that attenuation correction can increase TID to abnormal levels [26]. Thus MPI, even with attenuation correction, can miss balanced CAD.

### Temporal stability of results

In our referred population, interim analysis half-way into the study showed the same incidence of severe CAD in patients with a CAC score >1,000 and normal MPI (6 of 14 patients); the final combined proportion of patients with severe CAD was 15 of 26. Thus, among our patients with normal MPI and a CAC score >1,000, there was temporal stability in the proportion who had severe obstructive CAD. The confidence interval for the proportion of patients (57.7 %, 15 of 26) with severe CAD, normal MPI and CAC score >1,000 was 36.9 – 76.7 %. Larger studies and/or a meta-analysis of small studies are needed to more precisely estimate the CAD incidence in this population. However, this is the current best estimate.

### Cost effectiveness and clinical utility of a hybrid CAC score plus MPI study

MPI is well known to be cost effective, for example in reducing the number of cardiac catheterizations. A CAC score prior to MPI has recently been suggested to allow selection of patients at higher risk of ischemia [41] and may lead to fewer normal MPI examinations. However, it is known that a zero CAC score alone cannot rule out obstructive noncalcified CAD [42, 43]. The cost effectiveness and clinical utility of a routine hybrid CAC score plus MPI study needs to be determined.

### Conclusion

We prospectively determined the incidence of severe CAD in living patients with a very high CAC score and normal MPI in contrast to previous long-term retrospective studies investigating 10-year all-cause mortality. In symptomatic patients without known CAD a very high CAC score (>1,000) with a normal MPI result (high-risk stress markers, particularly TID, absent) was associated with an intermediate probability of severe CAD, typically requiring revascularization.

## Electronic supplementary material

ESM 1 (DOC 76 kb)
