# Supplementary material for: Very high coronary artery calcium score with normal myocardial perfusion SPECT imaging is associated with a moderate incidence of severe coronary artery disease
Source: Eur J Nucl Med Mol Imaging. 2015 Jul 4;42(10):1542–50. doi: 10.1007/s00259-015-3072-z (PMC4521098; doi:10.1007/s00259-015-3072-z)
Supplement: Supplementary file 1 — (DOC 76 kb) [file 259_2015_3072_MOESM1_ESM.doc]

**Note: references for APPENDIX 2-6 are at the end of the appendices**

**APPENDIX 1**

CT Coronary Artery Calcium Score Bibliography

Asymptomatic Patients

He ZX, Hedrick TD, Pratt CM, Verani MS, Aquino V, Roberts R, et al. Severity of coronary artery calcification by electron beam computed tomography predicts silent myocardial ischemia. Circulation 2000;101:244-51.

Hoff JA, Chomka EV, Krainik AJ, Daviglus M, Rich S, Kondos GT. Age and gender distributions of coronary artery calcium detected by electron beam tomography in 35,246 adults. Am J Cardiol 2001;87:1335-9.

Moser KW, O’Keefe JH, Bateman TM, McGhie IA. Coronary calcium screening in asymptomatic patients as a guide to risk factor modification and stress myocardial perfusion imaging. J Nucl Cardiology 2003;10:590-8.

Prognosis

Arad Y, Spadaro LA, Goodman K, Lledo-Perez A, Sherman S, Lerner G, et al. Predictive value of electron beam computed tomography of the coronary arteries: 19-month follow-up of 1173 asymptomatic subjects. Circulation 1996;93:1951-3.

Greenland P, LaBree L, Azen SP, Doherty TM, Detrano RC. Coronary artery calcium score combined with Framingham score for risk prediction in asymptomatic individuals. JAMA 2004;291:210-5.

Raggi P, Shaw LJ, Berman DS, Callister TQ. Prognostic value of coronary artery calcium screening in subjects with and without diabetes. J Am Coll Cardiol 2004;43:1663-9.

LaMonte MJ, Fitzgerald SJ, Church TS, et al. Coronary artery calcium score and coronary heart disease events in a large cohort of asymptomatic men and women. Am J Epidemiol 2005;162:421-9.

Nasir K, Shaw LJ, Liu ST, Weinstein SR, Mosler TR, Flores PR, et al. Ethnic differences in the prognostic value of coronary artery calcification for all-cause mortality. J Am Coll Cardiol 2007;50:953-60.

Raggi P, Gongora MC, Gopal A, Callister TQ, Budoff M, Shaw LJ. Coronary artery calcium to predict all-cause mortality in elderly men and women. J Am Coll Cardiol 2008;52:17-23.

Detrano R, Guerci AD, Carr JJ, Bild DE, Burke G, Folsom AR, et al. Coronary calcium as a predictor of coronary events in four racial or ethnic groups. N Engl J Med 2008;358:1336-45.

Becker A, Leber A, Becker C, Knez A. Predictive value of coronary calcifications for future cardiac events in asymptomatic individuals. Am Heart J 2008;155:154-60.

Blaha M, Budoff MJ, Shaw LJ, Khosa F, Rumberger JA, Berman D, et al. Absence of coronary artery calcification and all-cause mortality. J Am Coll Cardiol Img 2009;2:692-700.

Budoff MJ, Nasir K, McClelland RL, et al. Coronary calcium predicts events better with absolute calcium scores than age-sex-race/ethnicity percentiles: MESA (Multi-Ethnic Study of Atherosclerosis). J Am Coll Cardiol 2009;53:345-52.

Erbel R, Mohlenkamp S, Moebus S, Shaw LJ, Kinney GL, Chow D, et al. Coronary risk stratification, discrimination, and reclassification improvement based on quantification of subclinical coronary atherosclerosis: the Heinz Nixdorf Recall study. J Am Coll Cardiol 2010;56:1397-406.

Budoff MJ, Hokanson JE, Nasir K, et al. Progression of coronary artery calcium predicts all-cause mortality. JACC Cardiovasc Imaging 2010;3:1229-36.

Van Werkhoven JM, Schuijf JD, Jukema JW, Kroft LJ, Stokkel MP, bbets-Schneides P, et al. Anatomic correlates of a normal perfusion scan using 64-slice computed tomographic coronary angiography. Am J Cardiol 2008;101:40-5.

Ischemia

Rumberger JA, Sheedy PF, Breen JF, Schwartz RS. Electron beam computed tomographic coronary calcium score cutpoints and severity of associated angiographic lumen stenosis. J Am Coll Cardiol 1997;29:1542-8.

Guerci AD, Spadaro LA, Popma JJ, Goodman KJ, Brundage BH, Budoff M, et al. Relation of coronary calcium score by electron beam computed tomography to arteriographic findings in asymptomatic and symptomatic adults. Am J Cardiol 1997;79:128-33.

Lim E, Lahiri A. The importance of the link between coronary artery calcification and myocardial ischemia: a developing argument. J Nucl Cardiol 2007;14:272-4.

Rozanski A, Gransar H, Wong ND, Shaw LJ, Miranda-Peats R, Hayes SW, et al. Use of coronary calcium scanning for predicting inducible myocardial ischemia: Influence of patients' clinical presentation. J Nucl Cardiol 2007;14:669-79.

Berman DS, Shaw LJ, Hachamovitch R, Friedman JD, Polk DM, Hayes SW, et al. Comparative use of radionuclide stress testing, coronary artery calcium scanning, and noninvasive coronary angiography for diagnostic and prognostic cardiac assessment. Semin Nucl Med 2007;37:2-16.

Berman DS. Complementary roles of coronary calcium scanning and myocardial perfusion SPECT. J Nucl Cardiol 2004;11:379-81.

Schepis T, Gaemperli O, Koepfli P, Namdar M, Valenta I, Scheffel H, et al. Added value of coronary artery calcium score as an adjunct to gated SPECT for the evaluation of coronary artery disease in an intermediate-risk population. J Nucl Med 2007;48:1424-30.

LV Dysfunction

Budoff MJ, Shavelle DM, Lamont DH, Kim HT, Akinwale P, Kennedy JM, et al. Usefullness of electron beam computed tomography scanning for distinguishing ischemic from nonischemic cardiomyopathy. J Am Coll Cardiol 1998;32:1173-8.

Review

Nasir K, Clouse M. Role of nonenhanced multidetector CT coronary artery calcium testing in asymptomatic and symptomatic individuals. Radiology 2012;264:637-49.

**APPENDIX 2**

The 2,659 patients had the following characteristics: 1. Stable chest pain, 50% (1,318/2,659) and/or dyspnea (subjective or objective), 37% (973/2,659). 2. Abnormal prior test, 30% (798/2,659): exercise stress test 66% (523/798), echocardiogram 22% (177/798), coronary angiogram 5% (41/798), CAC score 0.5% (4/798), other abnormal test 36% (286/798). 3. Arrhythmia 10% (257/2,659). 4. Referred for preoperative assessment 4% (96/2,659). 5. Syncope 3% (89/2,659). Symptoms were chest pain (typical or atypical) and/or dyspnea. Dyspnea was either subjective (described by patient) or objective (determined by physician) due to pulmonary disease, deconditioning, obesity or angina equivalence.

On MPI, the prevalence of ischemia without MI on MPI was 8% (222/2,659). Abnormal MPI rate (MI and/or ischemia) was 11% (298/2,659). The distribution of CAC scores in our 2,659 patients was: CAC score 0: 24% (634/2,659), 1-10: 6% (165/2,659), 11-100: 18% (470/2,659), 101-400: 19% (514/2,659), 401-1,000: 16% (428/2,659), and >1,000: 17% (448/2,659). One-third of our 2,659 patients had a high CAC score of >400, 33% (876/2,659). The frequency of ischemia with a CAC score of <10 was very low, 0.5% (12/2,659). The frequency of ischemia with a CAC score of 0-100 was low, < 2% (1.5%, 39/2,659).

The distribution of CAC scores in the 222 patients with ischemia without MI was: CAC score 0: 3% (6/222), 1-10: 3% (7/222), 11-100: 12% (26/222), 101-400: 23% (51/222), 401-1,000: 27%, (60/222), and >1,000: 32% (72/222).

As expected, our 2,659 patients, referred for clinical reasons, have a different distribution of CAC scores compared to an asymptomatic population. Half of our patients had chest pain and one-third had dyspnea.

One-third of our 2,659 patients had a high CAC score of >400 whereas only 10% of the asymptomatic population of Budoff [*1*] had a high CAC score of >400. Our population had a greater incidence of a very high CAC score >1,000, 17% compared with Budoff’s asymptomatic population (N=25,253) where 4% had a very high CAC score.

Our 2,659 patients appear similar with respect to ischemia and MI prevalence and distribution of CAC scores, to studies that examined symptomatic patients. The prevalence of ischemia on MPI in our population was 8% similar to other papers, 6-11% [*2-3*]. In our study, half had chest pain as did Berman’s study 45% [*24]*, compared to Chang 17% [*4*] and Ho 15% [*5*]. The frequency of ischemia with a CAC score of <10 in our patients was very low, 0.5%. The frequency of ischemia with a CAC score of 0-100 was low, < 2% in our patients, similar to all the papers, <3% ( *2-3, 6*). The frequency of ischemia with a CAC score of >1,000 in our study was 16% similar or slightly higher than other papers, 9-15% [*4-8*]

The predictive value of the CAC score for ischemia is similar in all papers. We had an abnormal MPI rate (resting defect and/or ischemia) of 11% which is similar to the current incidence at another teaching hospital where 9% had an abnormal MPI result [*7*].

**APPENDIX 3**

Myocardial Imaging Technique

Patients were given written instructions on 24-h caffeine restriction or by telephone prior to arrival. The patients were interrogated on caffeine abstinence on the day of the stress test. Topical nitrates, worn for rest studies, were removed ½ -1 hour before the stress.

Rest followed by stress MPI was performed using technetium-99m sestamibi. Heavy patients (13/26), females >85 kg and males >100 kg, underwent a 2-day study with higher radiopharmaceutical doses of 1,000 to 1,800 MBq for rest and stress studies using a weight-based formula. Less heavy patients could undergo a 1-day study. 30 stops (frames) were acquired for SPECT. Imaging time per frame varied with body size 25-40 sec /frame for 1-day rest studies and 20-40 sec /frame for stress study. For 2-day studies, imaging time varied from 20-40 sec /frame with body size. For 2-day protocols, half of the studies of the small patients were acquired with half-time acquisition with depth-dependent resolution recovery. Images were examined with filtered back projection with and without attenuation correction as well as with iterative reconstruction with and without attenuation correction. Data acquisition was performed using combined SPECT and low dose CT scanning (GE Infinia Hawkeye) for attenuation correction and processed using both QGS/QPS and EC Toolbox software.

**APPENDIX 4**

Definition Of A Normal MPI

1. Visually normal MPI at rest and stress, and with <5% abnormality semi-quantitatively.
2. Normal left ventricular ejection fraction (LVEF) at rest (female/male) >50/45% [*9*].
3. In particular, a normal TID ratio of <1.2 [*10-11*], regardless of stress protocol except for very small left ventricles visually where TID ratio was ignored.
4. No decrease in LVEF of ≥7% after stress compared to rest.
5. No visual increase in right ventricular uptake with exercise only stress [*12*].
6. No visual increase in lung uptake with stress.
7. No relative hypotension with stress. A ≥10 mmHg systolic decrease with exercise or ≥30 mmHg systolic decrease with pharmacological stress alone was considered abnormal.
8. No high risk arrhythmia or other high risk ECG findings.

9. No chest pain with exercise without pharmacological stress.

10. No pallor, severe unexpected dyspnea, or other high risk clinical findings.

11. No ischemic ECG changes: normal resting ECG with greater than 1 mm downsloping

ST depression in 2 or more adjacent leads.

12. Uninterpretable resting ECG e.g. bundle branch block or repolarization abnormality was

not an exclusion criteria.

13. Abnormal myocardium, semi-quantitatively involving <5% of the LV, was regarded as

normal according to reporting guidelines from the American Society of Nuclear

Cardiology (ASNC) [*13*].

14. Normalcy semi-quantitatively was confirmed visually by an experienced nuclear cardiologist, board certified in Nuclear Medicine, with >5 years’ experience, or certified in Nuclear Cardiology by ASNC.

**APPENDIX 5**

**Demographics of Patients with CAC Score < 1,000 (N=410)**

|  |  | All patients |
| --- | --- | --- |
|  |  | (n=410) |
| Male |  | 213 (52%) |
| Age | Mean ± SD | 63 + 10 |
|  | Range | 34-89 |
| Hypertension |  | 267(65%) |
| Diabetes |  | 87(21%) |
| Dyslipidemia |  | 266 (65%) |
| Family history |  | 302 (73%) |
| Smoking |  | 254 (62%) |
| Pre-test probability of CAD | Low | 176 (43%) |
|  | Intermediate | 178 (43.5%) |
|  | High | 58 (13.5%) |
| Reasons for referral | Chest pain | 223(54%) |
|  | Typical | 102(24.5%) |
|  | Atypical | 121(29.5%) |
|  | and/or (Subjective or objective dyspnea) | 187(46%) |
|  | Preoperative assessment with CP | 2(0.5%) |
|  | LBBB | 4(1%) |

(4) For your comparison of the characteristics of the n=26 and n=410 I was

able to compare via chi-square tests (except for age). I assumed there were

no missing values.

**APPENDIX 6**

Comparison of the characteristics of the *N*=26* and *N*=410** (chi-square)

| Age - (unpaired t-test) *P*=0.022, (non-parametric test) *P* =0.013 | Significant |
| --- | --- |
| Sex - *P* =0.183 | Non- significant |
| Hypertension - *P* =0.669 | Non- significant |
| Diabetes - *P* =0.810 | Non- significant |
| Dyslipidemia - *P* =0.125 | Non- significant |
| Family History- 23% vs 73% - *P* <0.001 | Significant |
| Smoking - 27% vs 62% - *P* <0.001 | Significant |
| High pre-test probability of CAD - *P* <0.001 | Significant |
| Chest pain - *P* =0.063 | Non- significant |
| Typical chest pain - *P* =0.269 | Non- significant |
| Atypical chest pain - *P* =0.334 | Non- significant |
| Dyspnea - *P* =0.003 | Significant |
| Pre-operative assessment - *P* =0.019 (Fisher's exact test because of small expected cell counts) | Significant |
| LBBB- *P* =0.240 (Fisher's exact test) | Non- significant |

**N* = 26 (Normal MPI and CAC >1,000)

***N*= 410 (Normal MPI and CAC< 1,000)

References

1. Budoff MJ, Shaw LJ, Liu ST, Weinstein SR, Mosler TP, Tseng PA, et al. Long-term prognosis associated with coronary calcification: observations from a registry of 25,253 patients. J Am Coll Cardiol 2007;49:1860-70.
2. Mandour Ali MA, Bourque JM, Allam AH, Beller GA, Waston DD. The prevalence and predictive accuracy of quantitatively defined transient ischemia dilation of the left ventricle on otherwise normal SPECT myocardial perfusion imaging studies. J Nucl Cardiol 2011;18:1036-43.
3. Anand DV, Lim E, Hopkins D, Corder R, Shaw LJ, Sharp P et al. Risk stratification in uncomplicated type 2 diabetes: prospective evaluation of the combined use of coronary artery calcium imaging and selective myocardial perfusion scintigraphy. Eur Heart J 2006;27:713-21.
4. Burkhard N, Herzog BA, Husmann L, Ruegg C, Burger C, Leschka S, et al. Coronary calcium score scans for attenuation correction of quantitative PET/CT 13N-ammonia myocardial perfusion imaging. Eur J Nucl Med Mol Imaging 2010;37:517-21.
5. Chang SM, Nabi F, Xu J, Peterson LE, Achari A, Pratt CM, et al. The coronary calcium score and stress myocardial perfusion imaging provide independent and complementary prediction of cardiac risk. J Amer Coll Cardiol 2009;54:1872-82.
6. Ali I, Ruddy TD, Almgrahi A, Anstett FG, Wells RG. Half-time SPECT myocardial perfusion imaging with attenuation correction. J Nucl Med 2009;50:554-62.
7. Anand DV, Lim E, Hopkins D, Corder R, Shaw LJ, Sharp P et al. Risk stratification in uncomplicated type 2 diabetes: prospective evaluation of the combined use of coronary artery calcium imaging and selective myocardial perfusion scintigraphy. Eur Heart J 2006;27:713-21.
8. Ho J, FitzGerald S, Stolfus L, Cannaday J, Radford N. Severe coronary artery calcifications are associated with ischemia in patients undergoing medical therapy. J Nucl Cardiol 2007;14:341-6.
9. Sharir T, Germano G, Kavanagh PB, Lai S, Cohen I, Lewin HC, et al. Incremental prognostic value of post-stress left ventricular ejection fraction and volume by gated myocardial perfusion single photon emission computed tomography. Circulation 1999;100:1035-42.
10. Abidov A, Bax JJ, Hayes SW, et al. Transient ischemic dilation ratio of the left ventricle is a significant predictor of future cardiac events in patients with otherwise normal myocardial perfusion SPECT. J Am Coll Cardiol 2003;42:1818-25.
11. Abidov A, Germano G, Berman DS. Transient ischemic dilation ratio: a universal high-risk diagnostic marker in myocardial perfusion imaging. J Nucl Cardiol 2007;14:497-500.
12. Xu Y, Arsanjani R, Clond M, Hyun M, Lemley M Jr, Fish M, et al. Transient ischemic dilation for coronary artery disease in quantitative analysis of same-day sestamibi myocardial perfusion SPECT. J Nucl Cardiol 2012;19:465-73.
13. Hendel RC, Budoff MJ, Cardella JF, Chambers CE, Dent JM, Fitzgerald DM, et al. ACC/AHA/ACR/ASE/ASNC/HRS/NASCI/RSNA/SAIP/SCAI/ SCCT/SCMR/SIR 2008 Key Data Elements and Definitions for Cardiac Imaging: A Report of the American College of Cardiology/American Heart Association Task Force on Clinical Data Standards (Writing Committee to Develop Clinical Data Standards for Cardiac Imaging). Circulation 2009;119:154-86.
